# Evaluation of Apical Sealing Ability of a Calcium Silicate–Based Root Canal Sealer nRoot SP Using Different Obturation Techniques

**DOI:** 10.1016/j.identj.2025.100916

**Published:** 2025-07-23

**Authors:** Xin Wang, Pengfei Zhang, Yueyi Yang, Keyong Yuan, Meiling Jing, Yujie Zhang, Wenduo Tan, Zhengwei Huang, Chenguang Niu, Rui Ma

**Affiliations:** a Department of Endodontics, Shanghai Ninth People’s Hospital, Shanghai Jiao Tong University School of Medicine, College of Stomatology, Shanghai Jiao Tong University, Shanghai, China; b National Clinical Research Center for Oral Diseases, National Center for Stomatology, Shanghai, 200011, China; c Shanghai Key Laboratory of Stomatology, Shanghai, 200011, China

**Keywords:** Apical sealing ability, Epoxy resin-based root canal sealer, iRoot SP, Root canal filling materials, Root canal obturation

## Abstract

**Objectives:**

Well-qualified obturation, especially in the apical region, is the key to successful root canal treatment. The objective of the current study was to evaluate and compare the apical sealing ability of the calcium silicate–based root canal sealer nRoot SP with AH Plus and iRoot SP using both single-cone (SC) and continuous wave condensation (CWC) techniques.

**Methods:**

In total, 102 extracted human anterior teeth were decoronated and 12 mm of each root was preserved. After root preparation, the teeth were randomly divided into 6 groups of 17 teeth each: AH Plus with the SC technique, AH Plus with the CWC technique, iRoot SP with the SC technique, iRoot SP with the CWC technique, nRoot SP with the SC technique and nRoot SP with the CWC technique. The apical sealing ability of the teeth was measured by dye penetrant examination and scanning electron microscopy (SEM). The data were analysed using student’s *t*-test or one-way ANOVA with Tukey’s *post hoc* analysis for multiple comparisons. The results were considered statistically significant if P < .05.

**Results:**

nRoot SP and iRoot SP combined with both SC and CWC techniques demonstrated less apical dye leakages than did the AH Plus groups (*P* < .001). However, nRoot SP and iRoot SP showed similar apical dye leakages in both the SC and CWC subgroups (nRoot SP/SC and iRoot SP/SC groups (*P* = .673); nRoot SP/CWC and iRoot SP/CWC groups (*P* = .959)). Meanwhile, the apical dye leakages of the 3 sealers used within the SC and CWC subgroups were not statistically significant (AH Plus/SC and AH plus/CWC (*P* = .072); iRoot SP/SC and iRoot SP/CWC (*P* = .231); nRoot SP/SC and nRoot/CWC (*P* = .081)). In addition, the microgaps in both the nRoot SP and iRoot SP groups were much smaller than those in the AH Plus groups. Finally, the gap widths in the SC subgroups were slightly larger than those in the CWC subgroups.

**Conclusion:**

The apical sealing ability of nRoot SP is similar to that of iRoot SP with the SC and CWC techniques. nRoot SP and iRoot SP demonstrate better apical sealing ability than AH Plus with the SC and CWC techniques.

## Introduction

The dental pulp can be infected as a result of dental caries, trauma, or ascending infections from the periodontal tissues.^1^ Root canal therapy is currently the most effective treatment for endodontics diseases.^2^ However, successful root canal treatment depends on proper cleaning and shaping of the root canal system, well-qualified obturation and appropriate coronal rehabilitation.^3^ Approximately 60% of all retreatment cases stem from reinfection caused by inadequate root canal filling.^4^ Apical microleakage allows the entry of many periapical tissue fluids, microorganisms and antigens.^5^ Therefore, strict and complete 3-dimensional sealing of the root canal system with gutta-percha and root canal sealers to prevent microleakage, especially in the apical region, is the key to successful root canal treatment.^6^

According to Grossmann, the ideal root canal sealer should have an excellent sealing ability and slow setting time to ensure adequate working time, dimensional stability and biocompatibility.^7^ The resin-based sealer AH Plus (Dentsply International) is widely used in clinical practice as the gold standard of root canal sealers in combination with the continuous wave condensation (CWC) technique.^8^ However, AH Plus has limitations, such as potential mutagenicity, cytotoxicity and inflammatory responses. iRoot SP (Innovative BioCreamix) is a representative calcium silicate–based sealer with favourable flowability, small particle size and no setting shrinkage. Thus, the manufacturer has recommended the single-cone (SC) technique for the use of iRoot SP.^9^ Furthermore, calcium silicate–based materials also exhibit excellent biocompatibility and biomineralisation, high induction of pulp cell differentiation and antibacterial activity.^10^ Nowadays, nRoot SP (Enpunuo Bio-technology), a relatively new premixed injectable calcium silicate–based sealer, is gradually gaining acceptance for clinical application. Recommended by the manufacturer, nRoot SP exhibits good biocompatibility, high X-ray attenuation and osteoconductive effects and forms chemical bonds with dentin. nRoot SP is also non-cytotoxic and, in contrast to iRoot SP, heat resistant, which ensures the effectiveness of root canal treatment at high temperature. To our knowledge, this newly launched sealer has not yet been extensively studied. Therefore, the characteristics of nRoot SP should be studied to facilitate evidence-based decisions in clinical treatments.

Most root canal obturation techniques utilize gutta-percha and sealer to seal the space between the gutta-percha and canal wall.^11^ As an example, the CWC technique is widely used to fill irregular root canals due to its filling homogeneity and 3-dimensional adaptability.^12^ Meanwhile, the SC technique has become increasingly popular due to the advantages of simple operation, high clinical efficiency and minimal damage to the root canal.^13^ Although extensive studies have evaluated the obturation quality with SC and CWC techniques, they have reached inconsistent conclusions.^14^ Therefore, there has been no general consensus on the apical sealing ability of calcium silicate–based and resin-based sealers with different obturation methods. Thus, further studies are necessary to fully compare the efficacy of the CWC and SC techniques.

Therefore, the objective of the current study was to evaluate and compare the apical sealing ability of the calcium silicate–based root canal sealer nRoot SP with AH Plus and iRoot SP with the SC and CWC techniques, respectively, by dye penetrant examination and scanning electron microscopy (SEM). The null hypothesis was that no difference existed in the apical sealing ability of AH Plus, iRoot SP and nRoot SP with both SC and CWC techniques when measured by dye penetrant examination and SEM.

## Materials and methods

### Sample selection

G*Power 3.1.9.6 (Universitat Dusseldorf) was employed for calculating the sample size. Based on the previous experimental data, α was set at 0.05 and the study power at 0.8. The minimum sample size for each group was determined as 15.

One hundred and two single-rooted permanent teeth were cleaned and then preserved in 0.9% saline for subsequent experiments. The inclusion criteria for the teeth were as follows: single-rooted permanent teeth with a minimum of 12-mm root lengths; root canal curvature less than 15°; and complete development of the apical foramen. Exclusion criteria were cracks, internal or external resorption, carious teeth, teeth with calcified canals and teeth with endodontic treatment.

### Root canal preparation

The teeth were decoronated by a diamond bur (TF-13, Mani), preserving 12 mm of each root. A 15# K-file (Dentsply Sirona) was then introduced into each canal until the tip was visible at the foramen, and the working length was set as 1 mm short of this length. The root canals were prepared to #35 and 0.04 taper using nickel-titanium M3-Pro endodontic files (Yirui). During preparation, the root canals were lubricated with ethylenediaminetetraacetic acid, and each time the instruments were changed, the canals were rinsed with 1% NaClO solution alternating with 0.9% saline. The final irrigation was applied with 5 mL 0.9% saline. Finally, the root canals were dried with absorbent paper points (Gapadent).

### Root canal obturation

The teeth were randomly divided into 6 experimental groups based on sealers and obturation techniques (each group, *n* = 17) as follows:

Group AH Plus/SC: AH Plus with the SC technique
Group AH Plus/CWC: AH Plus with the CWC technique
Group iRoot SP/SC: iRoot SP with the SC technique
Group iRoot SP/CWC: iRoot SP with the CWC technique
Group nRoot SP/SC: nRoot SP with the SC technique
Group nRoot SP/CWC: nRoot SP with the CWC technique

The components of the sealers are presented in Table 1.

**Table 1:** Components of the sealers in the study

Root canal sealers	Manufacturer	Components
AH-Plus	Dentsply International Inc., York, PA, USA	Paste A: bisphenol-A epoxy resin, bisphenol-F epoxy resin, calcium tungstate, ziroconium oxide, silica, iron oxide pigments Paste B: dibenzyldiamine,ami-noadamantane tricyclodecanediamine, calcium tungstate, zirconium oxide, silica silicone oil
iRoot SP	Innovative BioCreamix Inc., Vancouver, Canada	Tricalcium silicates, dicalcium silicates, calcium phosphate monobasic, calcium hydroxide, zirconium oxide, fillers, and thickening agents
nRoot SP	Enpunuo Bio-technology Co., Jiangxi, China	Calcium silicates, zirconium oxide, silicon oxide, cellulose, calcium inorganic salt

For groups using the SC technique, the sealer was injected slowly into the root canal until the canal was nearly full. A 10# K-file (Dentsply Sirona) was lifted up and down to distribute the sealer evenly. The master gutta-percha cone was then coated with a thin layer of sealer and slowly inserted into the canal with tug-back to the working length. Then, the excess gutta-percha was trimmed off with a heat carrier (B&L Biotech) 2 mm below the orifice and vertically compacted with a cold plugger (B&L Biotech).

For groups using the CWC technique, the master gutta-percha cone was coated with a thin layer of sealer and slowly inserted into the canal with tug-back to the working length. The heated carrier was set at 180°C with the tip of the carrier 5 mm from the apical gutta-percha cone. Subsequently, the middle and coronal portion of the canals were backfilled with softened gutta-percha using B&L-Beta (B&L Biotech) at 180 °C to 2 mm below the orifice and vertically condensed with a suitable plugger. Afterwards, the canal orifice was sealed with flowable resin composite (3M™ Filtek™ Z350XT).

After obturation, all teeth were stored at 37 °C and 100% humidity for 7 days for the sealers to set completely.

### Apical sealing analysis by dye penetrant examination

The teeth were evenly coated with nail polish 3 times on the entire tooth surface except for a 2-mm portion around the apical foramen. Subsequently, these prepared teeth were dried and immersed in 2% methylene blue solution (Hengye Zhongyuan Chemical) for 7 days at 37 °C and 100% humidity. Then, the teeth were thoroughly washed under running tap water for 30 minutes. The nail polish was scraped off with a scalpel and the teeth were then allowed to dry. Next, the teeth were dissected longitudinally through the apical foramen and observed under an oral surgery microscope (Leica) with ×16 magnification. The maximum linear depth of dye penetration was measured using Image J (National Institute of Health).

### Scanning electron microscopy

The 2 remaining samples from each group were cut longitudinally with an abrasive disk. These samples were polished, sprayed with gold and examined using an FEI Scios 2 HiVac scanning electron microscope (Focalplane Electron Instruments). The bonding conditions of the material and dentin wall in all 6 experimental groups were assessed at cervical, middle and apical levels at various magnifications (×100, ×1,000, ×2,500).

### Statistical Analysis

SPSS 27 software (IBM SPSS Statistics for Windows) was used to process all data. The results are expressed as mean ± standard deviation (x̄ ± s). According to the Shapiro-Wilk test, the data were normally distributed. Thus, the data were analyzed using Student’s *t*-test or one-way ANOVA with Tukey’s *post hoc* analysis for multiple comparisons. The results were considered statistically significant if *P* < .05.

## Results

### Dye penetrant examination with different root canal sealers

**Fig. 1:**
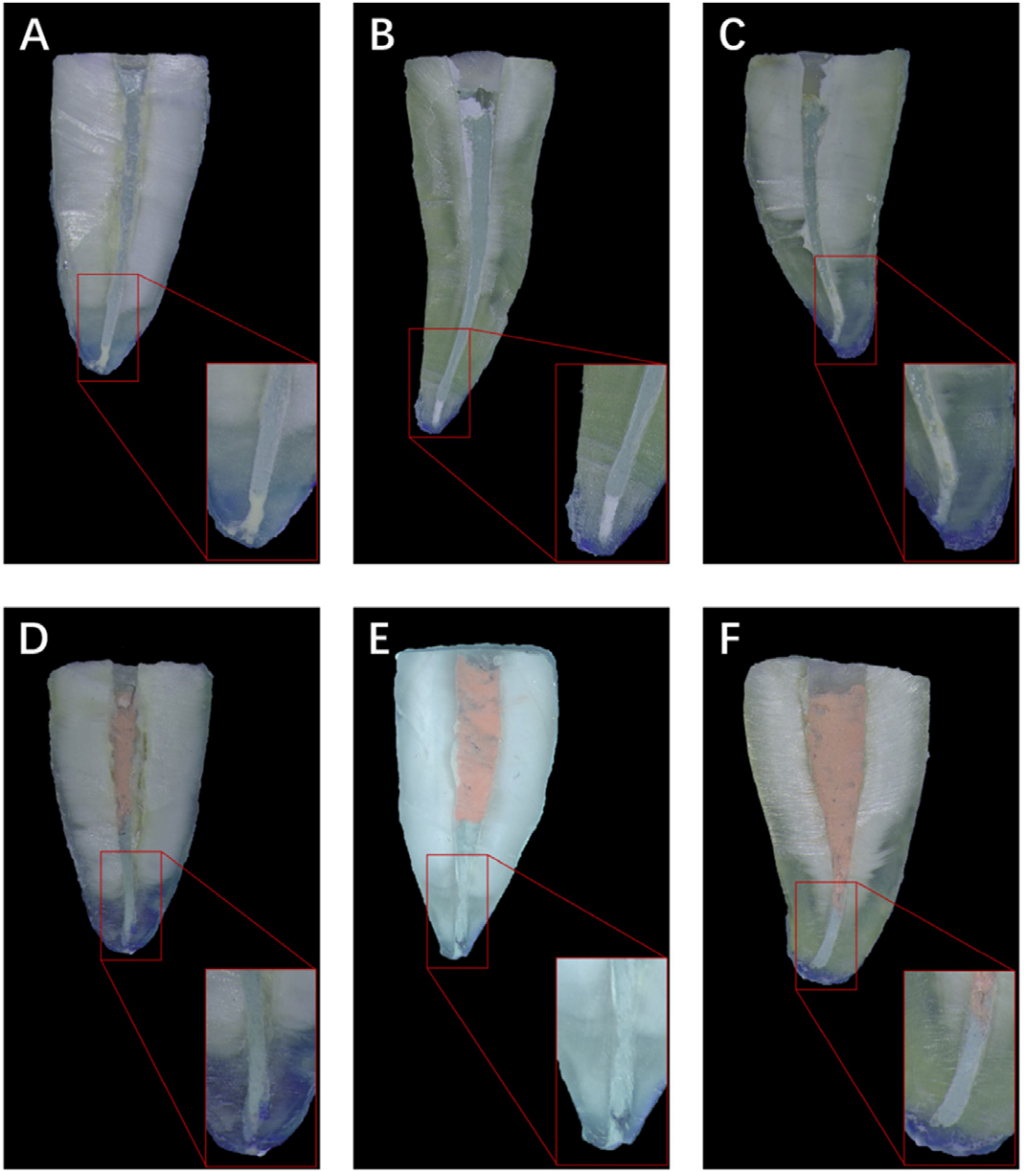
Representative images of apical microleakage. A, AH Plus/SC; B, iRoot SP/SC; C, nRoot SP/SC; D, AH Plus/CWC; E, iRoot SP/CWC; F, nRoot SP/CWC. CWC, continuous wave of condensation; SC, single-cone obturation.

Representative images of apical microleakages are presented in Figure 1.

The AH Plus groups demonstrated the highest apical microleakage. The data of apical dye leakages for 3 root canal sealers with SC and CWC techniques are shown in Table 2, Table 3. In the groups combined with SC technique, the apical dye leakage was significantly higher in roots filled with AH Plus compared with those obturated with iRoot SP and nRoot SP (*P* < .001). However, no statistically significant difference was found between iRoot SP/SC and nRoot SP/SC groups (*P* = .673). Meanwhile, in the groups combined with the CWC technique, the AH Plus group showed the highest mean leakage, while iRoot SP group showed the lowest. Likewise, the apical dye leakage of the AH Plus/CWC group was significantly higher than that of the other CWC subgroups (*P* < .001). However, there were no significant differences between iRoot SP/CWC and nRoot SP/CWC groups in terms of dye leakages (*P* = .959).

**Table 2:** Dye penetration length in the single-cone (SC) technique groups ($\bar{x}\pm s$, mm)

Group	Sample size	Dye penetration length	F	*P*	η^2^
AH Plus/SC	15	1.078 ± 0.098	21.517	<.001	
iRoot SP/SC	15	0.902 ± 0.067			0.506
nRoot SP/SC	15	0.926 ± 0.071			

In the groups combined with SC technique, the AH Plus subgroup showed higher dye penetration length than that in iRoot SP and nRoot SP subgroups (*P* < .001). The dye penetration length of the iRoot SP subgroup was similar to that of the nRoot SP subgroup (*P* = .673).

**Table 3:** Dye penetration length in the continuous wave condensation technique groups ($\bar{x}\pm s$, mm)

Group	Sample size	Dye penetration length	F	*P*	η^2^
AH Plus/CWC	15	1.007 ± 0.109	11.328	<.001	
iRoot SP/CWC	15	0.869 ± 0.078			0.350
nRoot SP/CWC	15	0.878 ± 0.075			

In the groups combined with CWC technique, the AH Plus subgroup showed higher dye penetration length than that in the iRoot SP and nRoot SP subgroups (*P* < .001). The dye penetration length of the iRoot SP subgroup was similar to that of the nRoot SP subgroup (*P* = .959).

### Dye penetrant examination with different obturation techniques

**Fig. 2:**
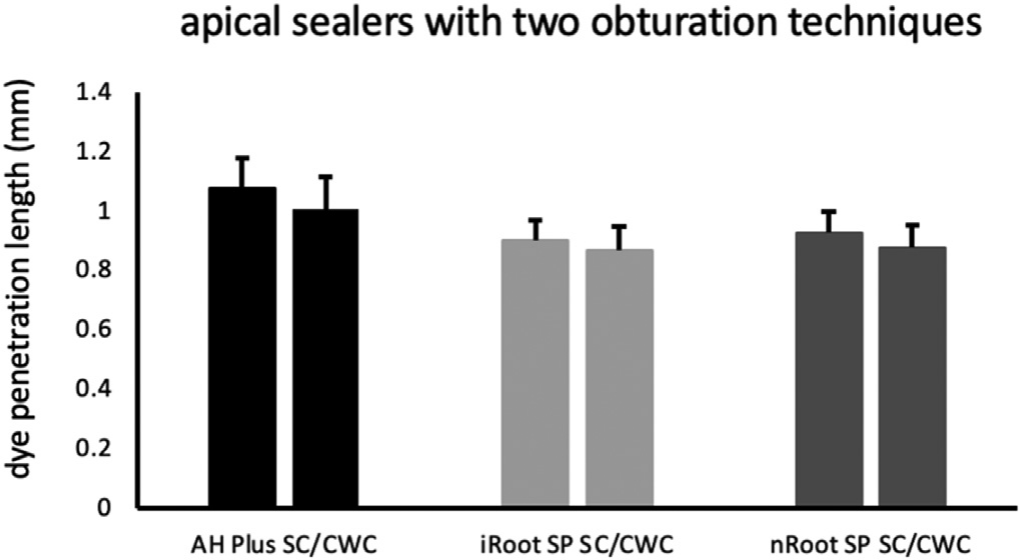
Dye penetration length of the two obturation techniques. CWC, continuous wave of condensation; SC, single-cone obturation. *P < .05.

Figure 2 demonstrates apical dye leakages for the 3 root canal sealers with the CWC and SC techniques. All root canal sealers exhibited higher dye leakage in the SC subgroups than the CWC subgroups, especially in the AH Plus groups. However, the differences of apical dye leakages between the 2 obturation techniques were not statistically significant (AH Plus/SC and AH plus/CWC (*P* = .072); iRoot SP/SC and iRoot SP/CWC (*P* = .231); nRoot SP/SC and nRoot/CWC (*P* = .081)).

### Scanning electron microscopy

**Fig. 3:**
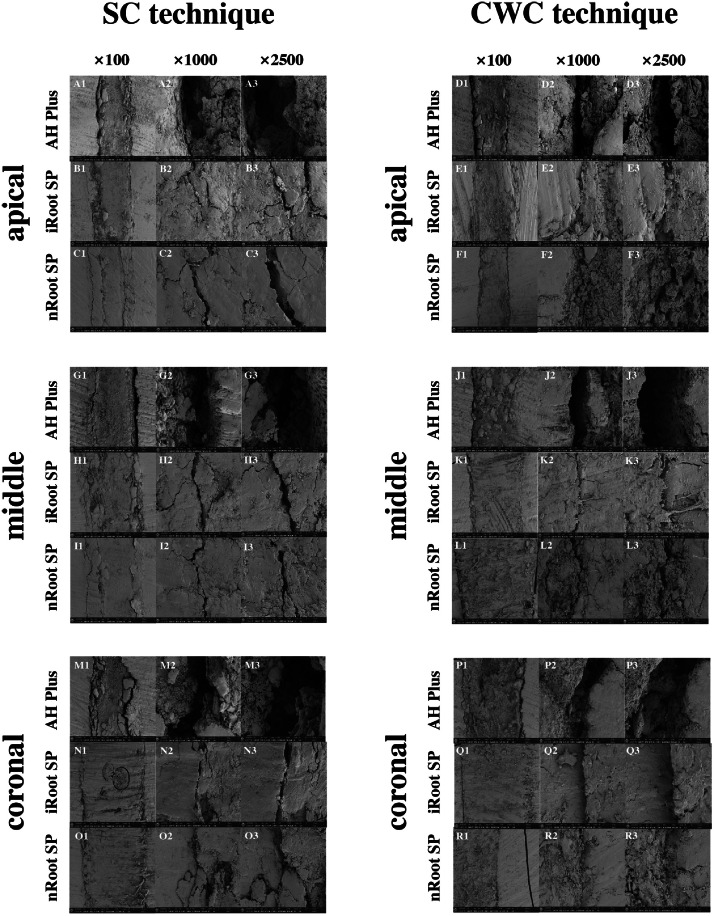
Scanning electron microscopy images of root canals in the coronal, middle and apical thirds with 3 different magnifications ([A1-R1] × 100, [A2–R2] × 1000, [A3–R3] × 2500) in the 6 groups.

SEM observations of the longitudinal sections are shown in Figure 3. Microgaps were seen at the root canal sealer–dentin bonding interface in the coronal, middle and apical thirds in all 6 groups, with the widest gap in AH Plus groups. The microgaps in both the iRoot SP groups and nRoot SP groups were much smaller than those in the AH Plus groups. Moreover, the gap widths in the SC subgroups were slightly larger than those in the CWC subgroups.

## Discussion

In this study, 3 root canal sealers were chosen, representing 2 different materials. To our knowledge, this is the first *in vitro* study on the apical sealing ability of the premixed calcium silicate–based sealer nRoot SP.

Dye penetrant examination is a simple technique that is widely used in apical microleakage studies. However, the results of the examination can be influenced by the dye and the immersion duration.^15^ In the current study, we found that within both the SC and CWC subgroups, the apical dye leakages were significantly higher in roots filled with AH Plus. This result is likely related to the smaller particle size, high flowability, lower film thickness and smoother surface of calcium silicate–based materials.^16^ Indeed, in the current study, nRoot SP and iRoot SP, which are both premixed calcium silicate–based sealers that have similar particle sizes, demonstrated similar apical sealing ability with the SC technique. Further, compared with the CWC technique, the SC technique only uses 1 gutta-percha cone and relies more on the root canal sealers.^17^ This may also explain why no significant difference was demonstrated between iRoot SP and nRoot SP with the SC technique. Similarly, Wang et al. demonstrated that iRoot SP achieved comparable filling quality and better dentinal tubules penetration than AH Plus with both the CWC and SC techniques.^18^ Meanwhile, Yu et al. evaluated the percentages of filled areas in the root canal and showed that, compared with epoxy resin–based sealer, bioceramic sealer demonstrated better filling quality with the SC technique.^17^

Similarly, no statistically significant difference was found between iRoot SP and nRoot SP groups with the CWC technique. According to a previous study, heating caused no detectable changes in the chemical structure of calcium silicate–based sealers, although microstructural changes may have occurred because of water loss, leading to increased porosity.^19^ Thus, it can be concluded in the current study that regardless of the obturation technique, nRoot SP has similar apical sealing ability as the representative calcium silicate–based sealer iRoot SP.

In the current study, when two obturation techniques using the same root canal sealer were employed, the apical microleakage values observed in groups with the SC technique were higher than those with the CWC technique. However, the difference was not statistically significant. This finding suggests that while the apical sealing efficacy of the CWC technique may be marginally superior to that of the SC technique, there appears no substantial distinction between their apical sealing performance. This finding is in general agreement with the studies conducted by numerous scholars.^20^, ^21^, ^22^, ^23^ Similarly, Angel et al. reported that iRoot SP and AH Plus both demonstrated similar penetration abilities into the lateral canals when using the SC and CWC techniques separately.^24^ Thus, whether the CWC or SC technique was employed, filling of the apical region still relied on sealers. In the current study, no statistically significant difference was demonstrated between the apical sealing ability of the SC and CWC techniques; consequently, it is necessary to improve the filling effect of the sealer in the apical root canal to ensure sufficient sealing ability.

SEM was also applied in this study because, compared with other apical microleakage assessment methods, its high magnification and excellent resolution demonstrate a submicron level of the junction zones, providing better observations of the surfaces of specimens.^25^ In the current study, SEM observations showed micro gaps in the junction zones of root canal sealers and dentin walls in the coronal, middle and apical thirds in all 6 groups. Among them, the AH Plus subgroups exhibited the widest gap, which was in line with some previous findings.^26^^,^^27^ The lower sealing ability of AH Plus shown with SEM could be explained by the incomplete polymerization and setting shrinkage of resinous components in AH Plus.^28^ Meanwhile, the iRoot SP and nRoot SP subgroups exhibited narrow gaps in the coronal, middle and apical thirds, displaying better sealing ability. However, no significant differences were found in gap width between the iRoot SP and nRoot SP groups. This finding may be explained by the excellent physical properties of the calcium silicate–based sealers, such as high flowability, good wettability, low film thickness, dimensional stability, hydrophilic tendencies and small particle size.^29^ Besides, the by-products produced by the calcium silicate–based sealers may have denaturized the dentin collagen fibers due to their alkaline nature, promoting the penetration of the root canal sealers.^30^ Moreover, in line with the dye penetrant examinations, the gap width in the SC subgroups was slightly larger than that in the CWC subgroups. However, SEM only provides 2-dimensional images that mainly focus on surfaces and are therefore unable to examine the internal structures of the samples. Besides, artificial gaps may also be created during the preparation of the samples, leading to the separation of the sealing materials from the dentin walls.^31^

## Conclusions

The calcium silicate–based root canal sealer nRoot SP combined with both the SC and CWC techniques demonstrated better performance in the apical sealing ability than AH Plus. However, there was no significant difference in the apical sealing ability among the nRoot SP and iRoot SP groups with the SC or CWC techniques. Moreover, no significant difference in the apical sealing ability between the SC and CWC techniques was found. Nonetheless, more *in vitro* and *in vivo* studies are required to confirm the properties of nRoot SP for broader clinical treatments.

## Conflict of interests

None declared.
